# Non-centralized and functionally localized nervous system of ophiuroids: evidence from topical anesthetic experiments

**DOI:** 10.1242/bio.019836

**Published:** 2017-04-15

**Authors:** Yoshiya Matsuzaka, Eiki Sato, Takeshi Kano, Hitoshi Aonuma, Akio Ishiguro

**Affiliations:** 1 Department of Physiology, Graduate School of Medicine, Tohoku University, 2-1 Seiryo-cho, Aoba ward, Sendai 980-8575, Japan; 2 Research Institute of Electrical Communication, Tohoku University, 2-1-1 Katahira, Aoba ward, Sendai 980-8577, Japan; 3 Research Institute for Electronic Science, Hokkaido University, Sapporo, Hokkaido 060-0812, Japan; 4 Japan Science and Technological Agency CREST, 4-1-8 Honcho, Kawaguchi, Saitama, 332-0012, Japan

**Keywords:** Ophiuroids, Locomotion, Non-centralized nervous system, Autonomous control, Functional localization

## Abstract

Ophiuroids locomote along the seafloor by coordinated rhythmic movements of multi-segmented arms. The mechanisms by which such coordinated movements are achieved are a focus of interest from the standpoints of neurobiology and robotics, because ophiuroids appear to lack a central nervous system that could exert centralized control over five arms. To explore the underlying mechanism of arm coordination, we examined the effects of selective anesthesia to various parts of the body of ophiuroids on locomotion. We observed the following: (1) anesthesia of the circumoral nerve ring completely blocked the initiation of locomotion; however, initiation of single arm movement, such as occurs during the retrieval of food, was unaffected, indicating that the inability to initiate locomotion was not due to the spread of the anesthetic agent. (2) During locomotion, the midsegments of the arms periodically made contact with the floor to elevate the disc. In contrast, the distal segments of the arms were pointed aborally and did not make contact with the floor. (3) When the midsegments of all arms were anesthetized, arm movements were rendered completely uncoordinated. In contrast, even when only one arm was left intact, inter-arm coordination was preserved. (4) Locomotion was unaffected by anesthesia of the distal arms. (5) A radial nerve block to the proximal region of an arm abolished coordination among the segments of that arm, rendering it motionless. These findings indicate that the circumoral nerve ring and radial nerves play different roles in intra- and inter-arm coordination in ophiuroids.

## Abstract (precis)

**Summary:** Functional localization exists in the nervous system of ophiuroids although there is no evidence of a morphological counterpart to the central nervous system of other species.

## INTRODUCTION

The neural mechanisms of locomotion in animals have been the focus of extensive studies in the field of neuroscience. Studies using vertebrates and arthropods have unveiled the autonomous as well as adaptive locomotor regulation by the interplay of reflex loops, central pattern generators and higher order centers such as cerebrum (McLean and Dougherty, 2015; Tuthill and Wilson, 2016). These neural structures are hierarchically organized, and each plays specific roles in locomotion (e.g. coordination of various body parts, generation of rhythmic patterned movements, initiation, modulation, and termination of gait).

In contrast to vertebrates and arthropods, little has been studied regarding the neural mechanism of locomotion in echinoderms, a phylum of marine animals that have a fundamentally different nervous system. Unlike other locomotory animals, they appear to lack a central nervous system that exerts centralized control over the usage of their body parts (Cobb and Stubbs, 1982). Their main nervous system is bipartite, consisting of (1) ectoneural components in the form of a circumoral nerve ring, which surrounds the mouth and is connected to five radial nerve cords each of which extends along an arm and whose neurons are aggregated into paired segmental ganglia, and (2) paired segmental hyponeural ganglia that are not connected longitudinally (Heinzeller and Welsch, 2001). There appears to be no hierarchical organization among these neural structures in contrast to those in vertebrates' locomotor control system.

In the present study, we investigated the neural mechanism underlying ophiuroids locomotion. Ophiuroids (brittle stars) locomote along the sea floor by coordinating the movement of five whip-like arms, each of which consist of multiple segments. Previous studies have described the characteristic patterns of arm movements during ophiuroid locomotion (Arshavskii et al., 1976a,b). A recent study that quantitatively analyzed arm movements during locomotion reported two representative types (Astley, 2012). In one type of locomotion, referred to as rowing, one arm that is in line with the direction of movement is pointed forward and functions as the center leading arm. The two adjacent arms, the left and right forelimbs, synchronously swing forward then backward cyclically to propel the disc. The two remaining arms, the left and right hind limbs, assist by driving the disc forward. In the other form of locomotion, referred to as reverse rowing, one pair of adjacent arms positioned in the direction of propulsion functions as the forelimbs. Among the remaining three arms, the two arms adjacent to the forelimbs function as the hindlimbs, while the arm positioned between the two hindlimbs functions as the center limb, which is pointed backward during locomotion. Reverse rowing is typically seen during the escape response (Yee et al., 1987). In both rowing and reverse rowing, the five arms which are structurally identical to each other play distinct roles in propelling the body in a particular direction. The forelimbs make large bilaterally symmetrical strokes. The hindlimbs make smaller strokes that are also bilaterally symmetrical. The center limb seems to contribute little to propulsion of the disc in both rowing and reverse rowing. Furthermore, the roles of each arm can be switched flexibly when the direction of locomotion is changed (Astley, 2012). The mechanisms by which a non-centralized, distributed nervous system determines the direction of locomotion, assigns different roles to individual arms, and initiates and synchronizes movements of the arms to perform coordinated movements are topics of interest in both neurobiological and computational sciences.

In the present study, we aimed to examine the underlying control mechanism of arm movements in ophiuroid locomotion. Our specific questions included:

1. What components of the nervous system are important for arm coordination during locomotion? In particular, we were interested in the role of the circumoral nerve ring because it forms the anatomical basis through which the radial nerve of each arm interacts with those of the other arms (Cobb and Stubbs, 1981, 1982).
2. What are the underlying control mechanisms of locomotion? More specifically, how do ophiuroids assign specific roles to their arms? Despite anatomical similarity among the five arms, each arm plays a different role in locomotion. How does an ophiuroid determine which arm acts as the center limb, forelimb, or hindlimb?

In order to study these issues, we applied selective anesthesia to various body parts to examine its effect on locomotion. Because gait control is intimately dependent on the dynamics of the limbs (Full and Koditschek, 1999), we blocked nerve conduction by topical application of L-menthol (Costello et al., 1957; Costello and Henley, 1971), which preserved the dynamics of the body.

## RESULTS

### Locomotion in intact subjects

**Fig. 1.**
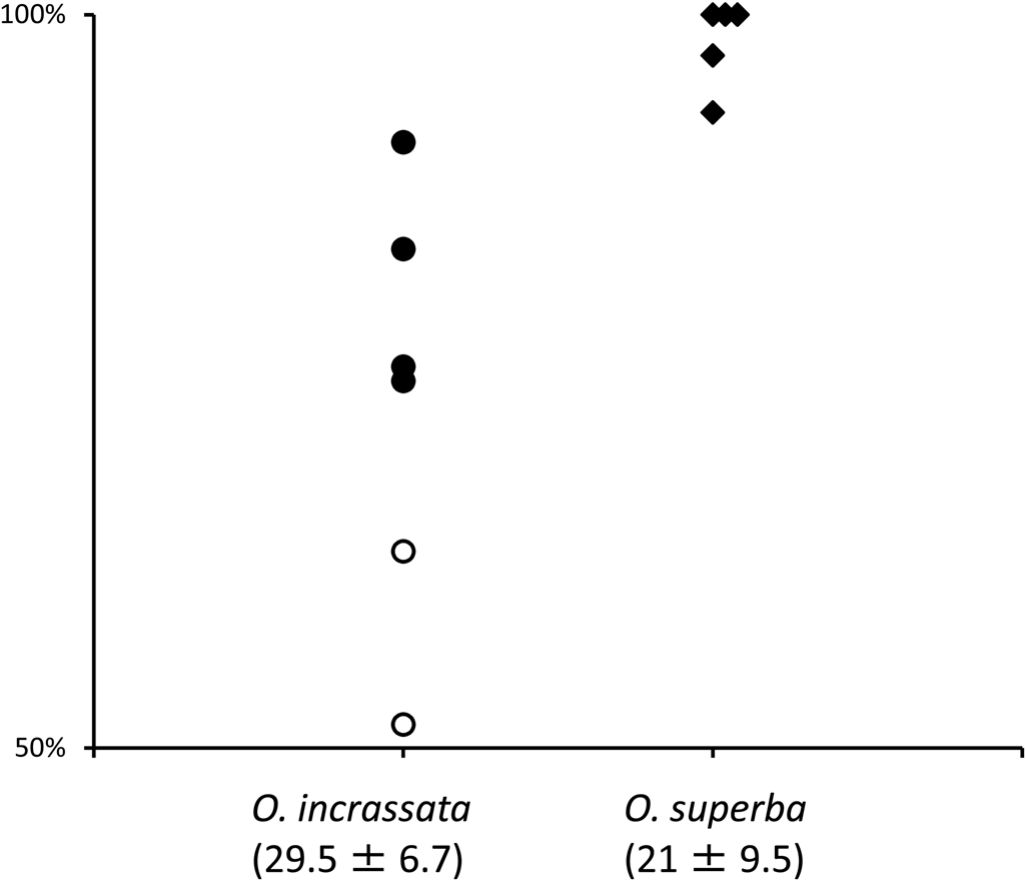
**Occurrence of different types of locomotion in intact ophiuroids.** Percentage of rowing movements are indicated for individual subjects of *O.incrassata* (circles, *n*=6) and *O. superba* (diamonds, *n*=5). The numbers in parentheses are the mean and s.d. of the number of movements observed within 30 min. Except for the two subjects of *O. incrassata* (open symbols), all the subjects performed rowing movements significantly more often than reverse rowing movements (*P*<0.01 by chi square test).

**Fig. 2.**
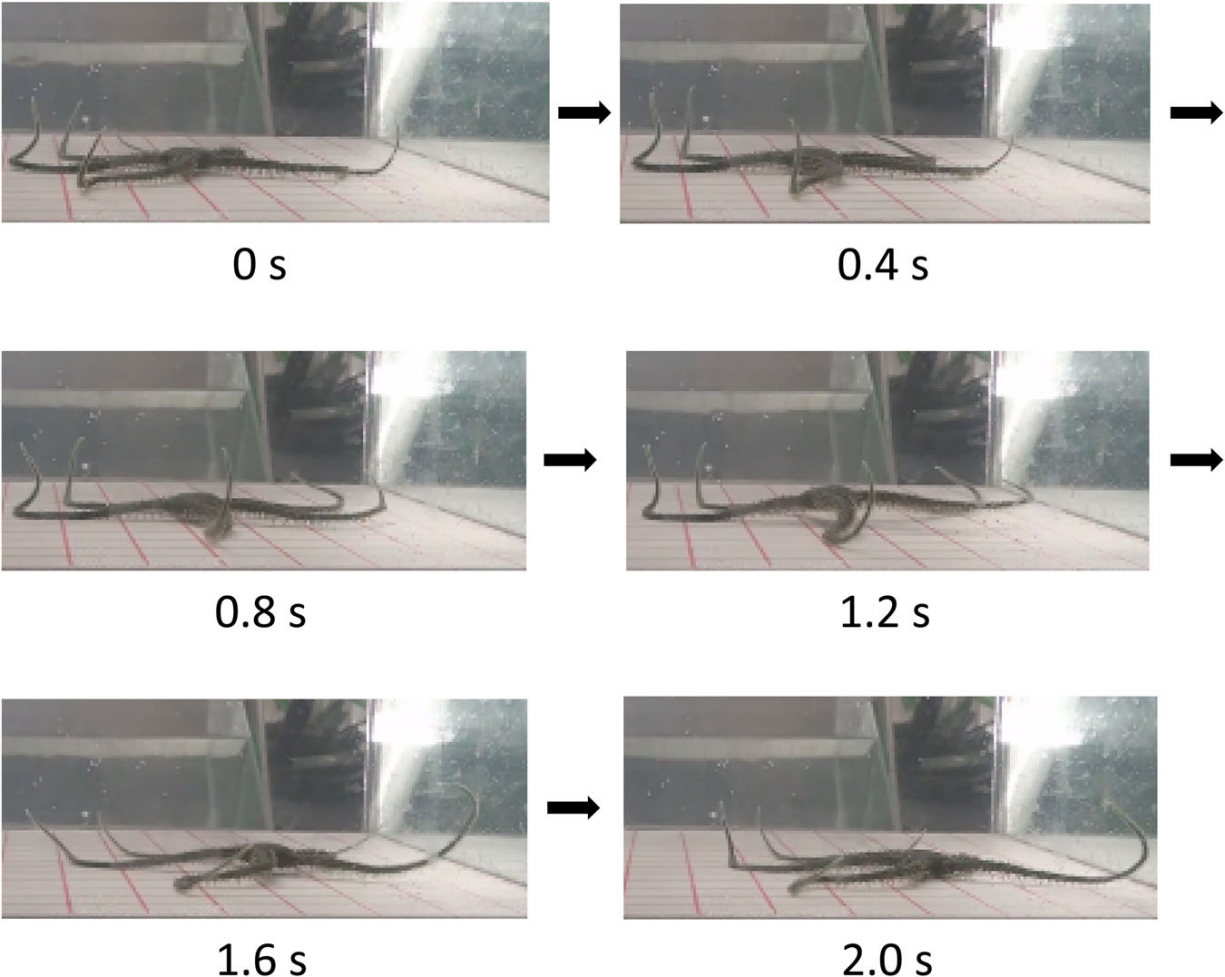
**Side view during rowing.** An *O. incrassata* viewed from the side, while advancing to the right of the figure. In the first three images (0, 0.4, and 0.8 s) the disc remains motionless on the floor, while the forelimbs are swung forward. In the next three images (1.2, 1.6, and 2 s) the disc is elevated from the floor and then propelled forward by the backward stroke of the forelimbs. Note that the midsegments of the forelimbs are in contact with the floor, while the disc is elevated (1.2 and 1.6 s).

We first examined whether intact brittle stars preferred to perform rowing or reverse rowing movements, which have been the two types of locomotion typically reported. All the subjects from both species preferred rowing over reverse rowing, which is consistent with the findings of previous studies (Fig. 1) (Arshavskii et al., 1976a; Astley, 2012). Four of six *O. incrassata* and five *O. superba* performed rowing significantly more frequently than reverse rowing movements (*P*<0.01 by chi square test). In two *O. incrassata*, the difference was not significant. In addition to rhythmic arm movements in a horizontal plane, which have been previously reported (Arshavskii et al., 1976a,b; Astley, 2012), we observed that the disc and arms also performed rhythmic vertical movements (Fig. 2). A side view of ophiuroid locomotion revealed that the forelimbs were lifted off the floor during the forward stroke, while the disc maintained contact with the floor. In contrast, during the backward stroke of the forelimbs, the midsegments of the arms made contact with the floor, while the disc was elevated from the floor before being thrust forward. The distal segments of the arms were bent aborally throughout locomotion; thus, they made no contact with the floor.

### Anesthesia of the disc

We examined the effects of anesthesia of the disc in two *O. incrassata* subjects. We applied powdered menthol on the oral surface of the disc for two minutes, then washed it away and returned the subjects to the tank filled with artificial seawater. Care was taken to avoid spillage of the powder into the oral cavity.

**Fig. 3.**
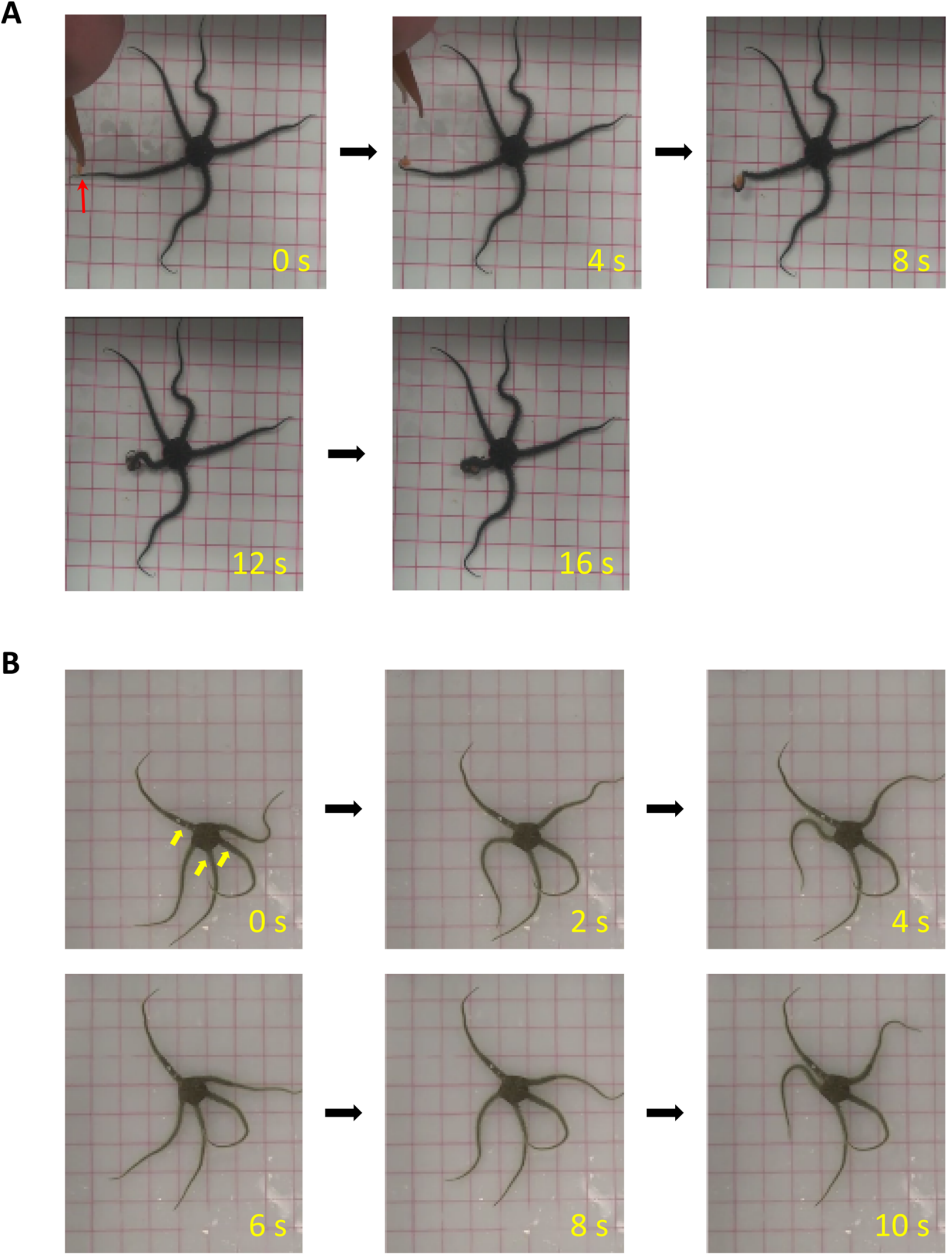
**Effects of nerve blocks of the circumoral ring and proximal radial nerves.** (A) Anesthesia of the circumoral ring completely immobilized ophiuroids. The anesthetized subjects showed no spontaneous arm movements in the tank. When food (dried shrimp, red arrowhead at 0 s) made contact with one of the arms, it was promptly captured and relayed to the disc (4–16 s). Red horizontal and vertical lines represent 2 cm intervals. See Movie 1 for continuous movement. (B) Radial nerves of three arms of this ophiuroid were damaged by a fine point drill at the proximal segments (indicated by yellow arrowheads at 0 s). Intact arms made whip-like swinging movements that consisted of flexion and extension of the segments, but arms with damaged nerves showed no spontaneous movements. Note that the arms with damaged nerves maintained similar curvature throughout the locomotion (0–10 s).

Following the induction of anesthesia, anesthetized subjects were unable to perform either rowing or reverse rowing movements. Unlike intact subjects, physical touch to the arms and disc of anesthetized subjects failed to elicit escape movements. To ascertain that the unresponsiveness was not due to dispersion of the anesthetic agent to the arms through the water vascular system, we placed a food morsel (a piece of dried shrimp) in contact with one of the arms. The subject grasped the food with that particular arm and attempted to bring it to the mouth. Nevertheless, ingestion was not possible owing to paralysis of the jaw (Fig. 3A; Movie 1). This observation indicates that motility of the arms was preserved even after anesthesia of the circumoral nerve ring.

Anesthesia of the disc was likely to have also anesthetized the proximal portions of the radial nerves. Therefore, to investigate the effects of selective lesions on the proximal radial nerves while leaving the circumoral nerve ring intact, we inflicted surgical lesions on the radial nerves at the base of the arms by a drill (see Materials and Methods). Following a recovery period of 1 h, the operated subjects were unable to initiate locomotion, similarly to the subjects in which the disc was anesthetized. In one subject, we compared the motility of arms with lesions with that of intact arms. We found that lesions on the proximal radial nerve abolished coordinated movements of the segments. As a result, the arms with lesions did not perform the whip-like movements typical of intact arms during locomotion (Fig. 3B). Nevertheless, subjects were able to grasp food and bring it to the disc when the morsel made contact with the distal portions of the lesioned arms.

### Anesthesia of five radial nerves

We examined whether functional differences exist among various segments of the radial nerves. For this purpose, we applied anesthesia to specific areas of the arms. We compared the effects of anesthesia of the midsegments and distal segments of the five arms. Because the manner in which these portions of the arms made contact with the floor during locomotion differed (Fig. 2), we expected them to generate different types of sensory signals during locomotion.

**Fig. 4.**
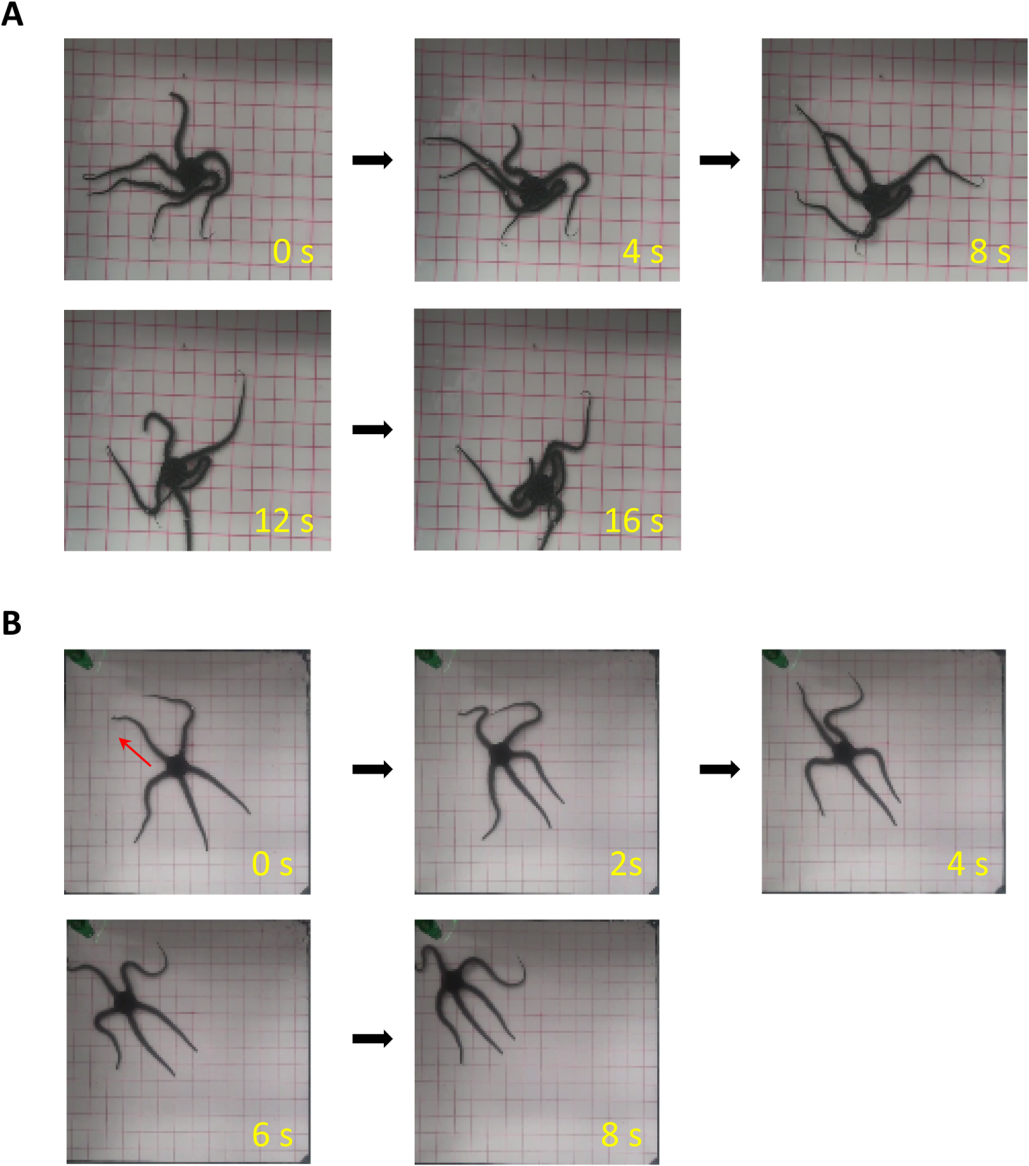
**Effects of anesthetizing midsegments of all five arms.** (A) A subject (*O. incrassata*) in which the midsegments of five arms were anesthetized. Following anesthesia, the five arms maintained spontaneous movements; however, movements were completely uncoordinated. The subject was unable to move in any consistent direction. See also Movie 2. (B) A subject in which the distal segments of five arms were anesthetized. The subject made rowing movements even after administration of anesthesia, to move from the center to the corner of the tank. Red arrow indicates the direction of locomotion.

We first anesthetized the midsegments of the five arms with L-menthol for 5 min. In contrast to the subjects in which the circumoral nerve rings were anesthetized, subjects under anesthesia of the radial nerves showed no loss of voluntary movements in the arms. However, when the anesthetized subjects were returned to the experimental tank, they were no longer able to perform coordinated arm movements for locomotion. The five arms moved asynchronously, and any particular pair of arms did not make symmetric swinging movements as in rowing movements. Thus, the arm movements did not generate force to propel the disc in any consistent direction. As a result, the anesthetized subjects often haphazardly changed the direction of locomotion and sometimes even strayed from the corner towards the center of the tank, against their natural tendency to avoid open spaces (Fig. 4A; Movie 2).

We also examined the effects of anesthesia of the distal segments of the arms. In contrast to the subjects under anesthesia of the midsegments, these subjects showed no difficulty in making coordinated arm movements (Fig. 4B), and were able to perform rowing and reverse rowing movements even after anesthesia.

### Effects of amputation of anesthetized arms

The midsegments of the arms periodically touched the floor during locomotion and thus generated periodic contact sensations. The importance of this periodic sensory feedback was demonstrated by the fact that anesthesia of these segments in all arms completely disrupted the coordination of arm movements for locomotion. This finding raised the question of whether periodic sensory feedback must be specifically generated by the midsegment, or by any other segment of the arms. To examine this further, we anesthetized the midsegment of each arm and then amputated the arms at a point proximal to the anesthetized portion. Thus, remnants of the arms regained contact with the floor.

**Fig. 5.**
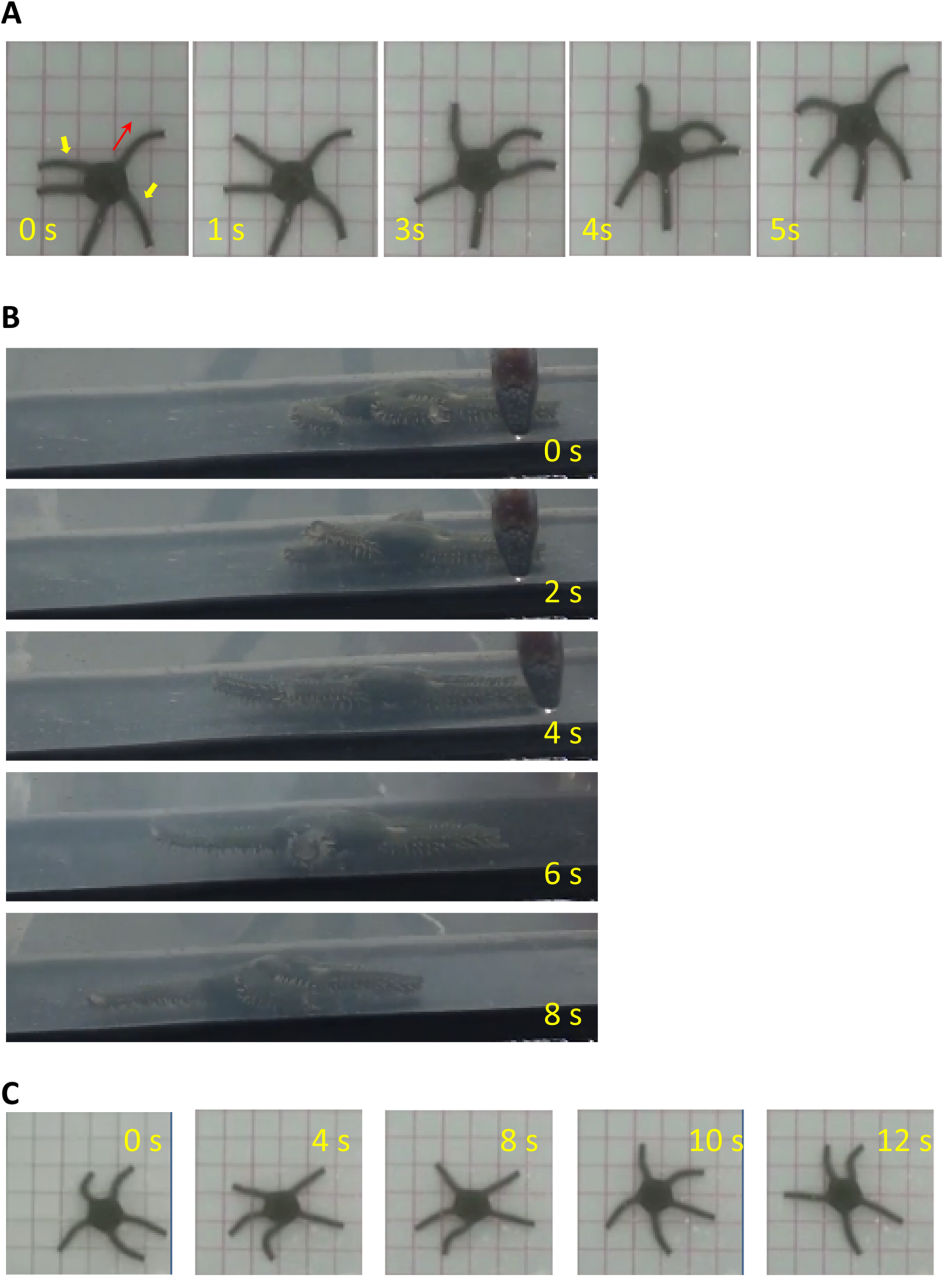
**Effects of amputation of anesthetized arms.** (A) A subject (*O. incrassata*) whose arms were amputated following anesthesia. Anesthesia of the midsegments of five arms rendered this subject unable to coordinate arm movements for locomotion (Fig. 4A; Movie 2). Following amputation of the arms proximal to the anesthetized portions, this subject resumed rowing movements using a pair of arm stubs (indicated by yellow arrows) as forelimbs (0–8 s). Red arrow indicates direction of locomotion. (B) Side view of the same subject during locomotion. The subject is advancing to the left of the figure. Arm stubs, which acted as forelimbs periodically touched the floor as the disc advanced forward (6–8 s). (C) After the amputation, the distal ends of the arm stubs were anesthetized by L-menthol. The subject failed to coordinate arm movements for rowing movements. See Movie 3.

Following anesthesia, the subject was again unable to coordinate arm movements for locomotion, and failed to move consistently in any particular direction. After amputation, however, the subject (i.e. the central disc and arm stubs) regained the ability to locomote (Fig. 5A). Furthermore, movements of the arm stubs were similar to typical rowing movements, i.e. one arm stub was pointed in the direction of locomotion, and its adjacent arm stubs synchronously and cyclically made forward and backward strokes. Moreover, observation from the side revealed that the ends of the forelimbs stubs made contact with the floor during the backward stroke and were lifted from the floor during the forward stroke similarly to the forelimbs of intact ophiuroids (Figs 2 and 5B). Finally, in order to deprive the subject of the contact sensation, we anesthetized the distal ends of the arm stubs that made contact with the floor during the restored locomotion. The anesthesia impaired the coordination of arm movements for rowing movements. Consequently, the subject was again unable to move straightly (Fig. 5C; Movie 3).

### Anesthesia of the radial nerves of selected arms

The importance of sensory input to the arms during locomotion became evident from the aforementioned findings. Observation of intact subjects indicated that periodic contact of the midsegments with the floor was the major source of sensory stimuli (Fig. 2). Therefore, in the experiments that followed, we systematically varied the number of arms in which the midsegments were anesthetized and examined the manner in which anesthesia affected locomotion. Specifically, we examined: (1) whether anesthetized subjects were still capable of making coordinated arm movements; (2) whether rowing or reverse rowing movements predominate following anesthesia; and (3) the manner in which anesthetized arm(s) acted differently from intact arms.

#### Anesthesia of one arm

**Fig. 6.**
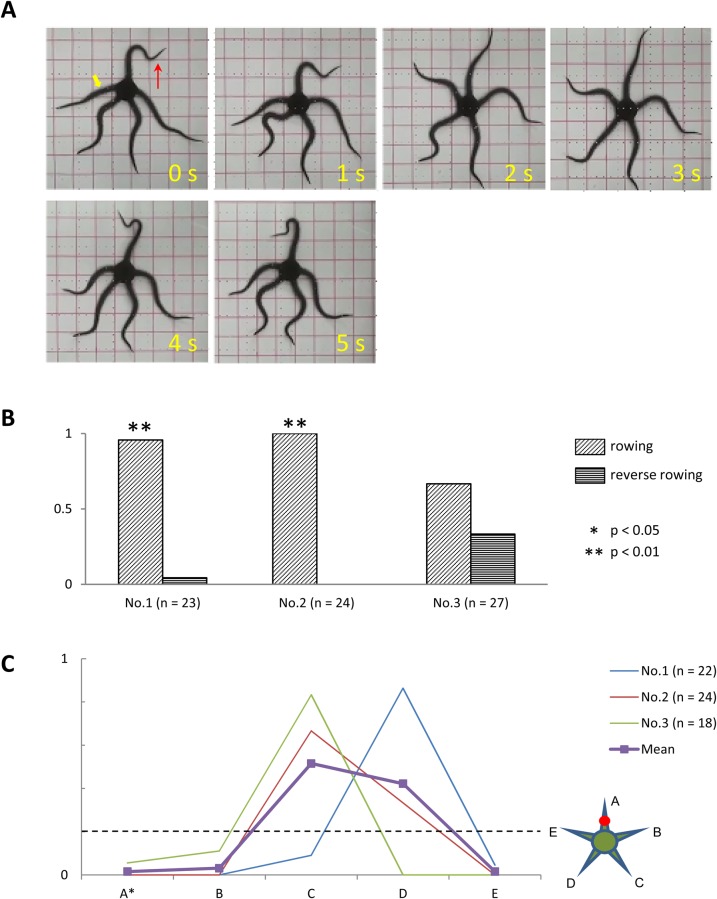
**Effects of anesthesia of one arm.** (A) Rowing movement of a subject (*O. incrassata*), in which the midsegment of one arm was anesthetized. Images taken at 1-s intervals. In the first image, the anesthetized arm is indicated by the yellow arrowhead and the direction of locomotion is indicated by the red arrow. The subject was able to perform rowing movements without difficulty. (B) Frequency of rowing (obliquely hatched bars) and reverse rowing (horizontally hatched bars) movements in subject Nos. 1–3. Numbers in parentheses indicate the total number of rowing and reverse rowing movements observed for each subject. Asterisks indicate significant differences in frequency (*P<0.05, **P<0.01, by chi-square test). (C) Frequency at which each arm acted as the leading arm. Results from individual subjects (Nos. 1–3) are represented by different colors. The thick line with square markers represents the average of all subjects. The horizontal dashed line indicates the chance level frequency (=0.2). Numbers in parentheses indicate the number of rowing movements observed for each subject. Inset illustrates the aboral view of the spatial arrangement of the anesthetized and intact arms. The anesthetized arm is designated arm A (marked by an asterisk in the abscissa), whereas the intact arms are designated B–E, clockwise.

In three subjects, we examined the effects on locomotion of anesthetizing one arm at the midsegments. In contrast to the subjects in which all arms were anesthetized, subjects with only one arm anesthetized promptly moved towards the corner when placed in the center of the tank. Furthermore, well-coordinated rowing movements were observed in these subjects while moving towards the corner of the tank (Fig. 6A). Similarly to the intact subjects, the anesthetized subjects performed rowing movements far more frequently than reverse rowing movements (Fig. 6B). With the exception of subject No. 3, rowing movements were significantly more frequent than reverse rowing movements (*P*<0.05). We also examined the roles of anesthetized and intact arms during rowing. We found that ophiuroids tended to use intact arms that had not been anesthetized as the leading arm (Fig. 6C). Among the four intact arms, one of the two that were furthest from the anesthetized arm acted as the leading arm more frequently than any of the two intact arms adjacent to the anesthetized arm. In contrast, the anesthetized arm acted as the leading arm very infrequently. In one subject (No. 3), the anesthetized arm acted as the leading arm in just 5.6% of all rowing movements observed. In the remaining subjects, the anesthetized arm never once acted as the leading arm.

#### Anesthesia of two adjacent arms

**Fig. 7.**
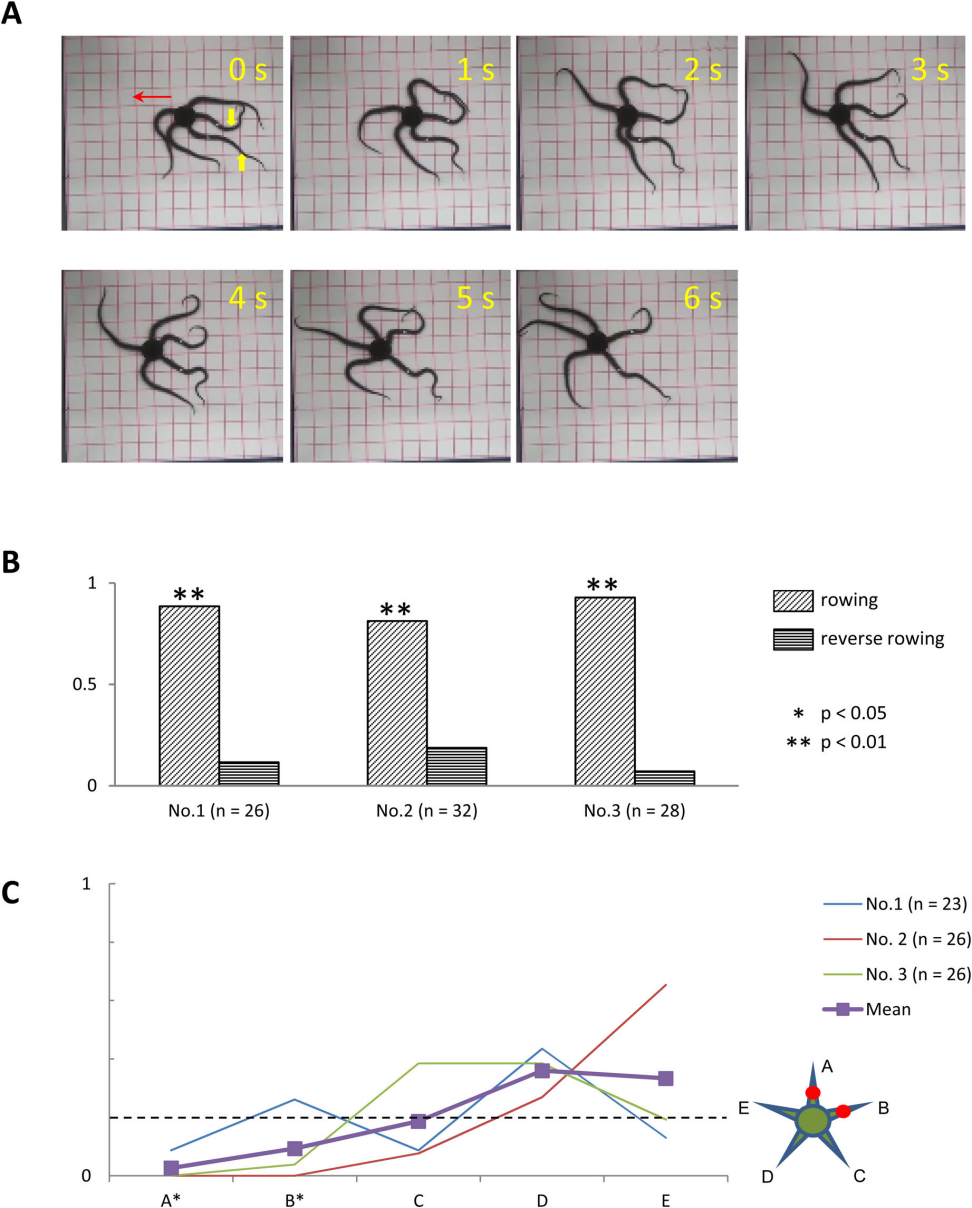
**Effects of anesthesia of two adjacent arms.** (A) Rowing movement of a subject (*O. incrassata*), in which the middle segments of two adjacent arms were anesthetized (indicated by yellow arrowheads). (B) Frequency of rowing and reverse rowing movements in subject Nos. 1–3. (C) Frequency at which each arm acted as the leading arm. Anesthetized arms are designated A and B, whereas intact arms are designated C–E, clockwise. Other conventions are the same as in Fig. 6.

In three subjects (two *O. incrassata* and one *O. superba*), we examined the effects of anesthetizing two adjacent arms at the midsegments on locomotion. The anesthetized subjects were able to perform coordinated arm movements to move from the center to the corner of the tank (Fig. 7A). In addition, rowing movements in all subjects were significantly more frequent than reverse rowing movements (Fig. 7B). As in the previous experiment, subjects mainly used the intact arm as the leading arm (Fig. 7C). In contrast, anesthetized arms very infrequently acted as leading arms. In subject Nos. 1 and 3, both anesthetized arms acted as leading arms below the expected chance level. In subject No.2, one of the anesthetized arms acted as the leading arm at the chance level (26% of observed rowing movements), whereas the other anesthetized arm did so below chance level.

#### Anesthesia of three adjacent arms

Our experiments thus far indicated that ophiuroids perform rowing movements by default even when some of the arms are anesthetized. In addition, a previous study demonstrated that rowing movements require a minimum of three intact arms (Arshavskii et al., 1976b). This drew our attention to the manner in which ophiuroids would locomote if anesthesia left less than three arms intact. To address this question, we anesthetized three adjacent arms and examined the effects on locomotion in four subjects (three *O. incrassata* and one *O. superba*).

**Fig. 8.**
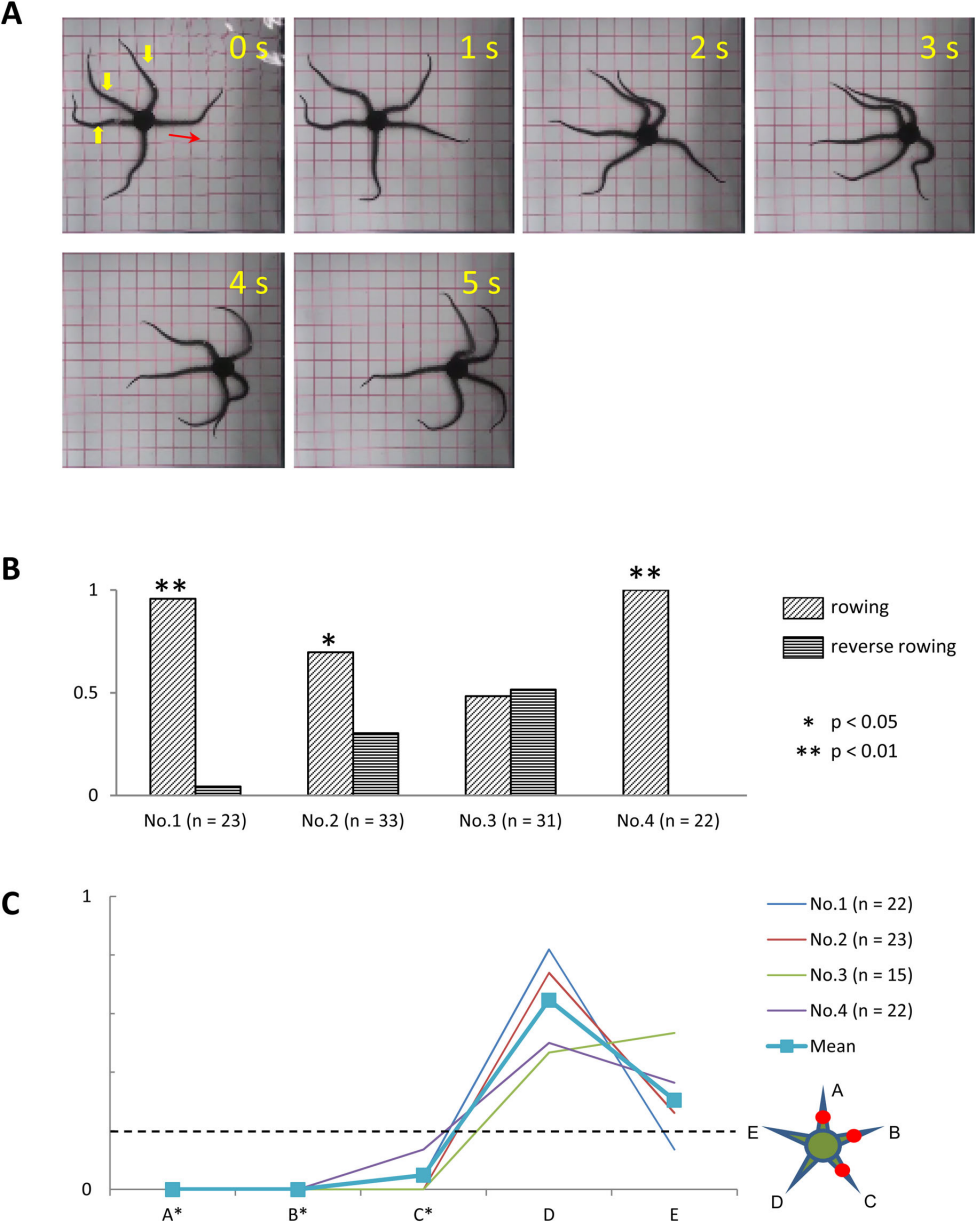
**Effects of anesthesia of three adjacent arms.** (A) Rowing movement of a subject (*O. incrassata*), in which the middle segments of three adjacent arms were anesthetized (indicated by yellow arrowheads). (B) Frequency of rowing and reverse rowing movements for subject Nos. 1–4. (C) Frequency at which each arm acted as the leading arm. Anesthetized arms are designated A, B and C, whereas intact arms are designated D–E, clockwise. Other conventions are the same as in Fig. 6.

We found that the subjects in which three adjacent arms were anesthetized were still able to perform rowing movements (Fig. 8A). While rowing, they used one of the two intact arms as the leading arm, the other intact arm as a forelimb, and an anesthetized arm as the other forelimb. The movement of the anesthetized forelimb, however, differed from that of the intact forelimb in two ways. First, the anesthetized forelimb was rendered inflexible. While rowing, approximately 2 cm of its midsegment remained straight as it swung either forward or backward. In contrast, the intact forelimb flexed smoothly like a whip as it swung in motion. Second, the proximal portion of the anesthetized forelimb swept over a larger angle than the intact forelimb to compensate for the impaired mobility of its anesthetized segments.

With the exception of one subject (No. 3), rowing movements were performed significantly more frequently than reverse rowing movements (Fig. 8B). Furthermore, as in the preceding experiments, leading arms during rowing were mostly the intact arms (Fig. 8C). When reverse rowing movements did occur, particularly in subjects No.2 and No.3, the two intact arms always acted as forelimbs while the anesthetized arms acted as hindlimbs and center limb.

#### Anesthesia of two separate arms

In the preceding experiments, anesthetized subjects were still able to make coordinated movements of their arms while rowing. In the above experiments, however, anesthesia left multiple adjacent arms intact. The question arose of whether coordination between arms is still preserved if intact arms are interspersed with anesthetized arms. To address this issue, we anesthetized the midsegments of two arms that were separated by one intact arm, and examined the effects in four subjects (three *O. incrassata* and one *O. superba*). This left one isolated arm and two arms adjacent to each other intact. If any of the intact arms functioned as the leading arm, then the leading arm and the hindlimbs would be separated by anesthetized arms, which would function as the forelimbs.

**Fig. 9.**
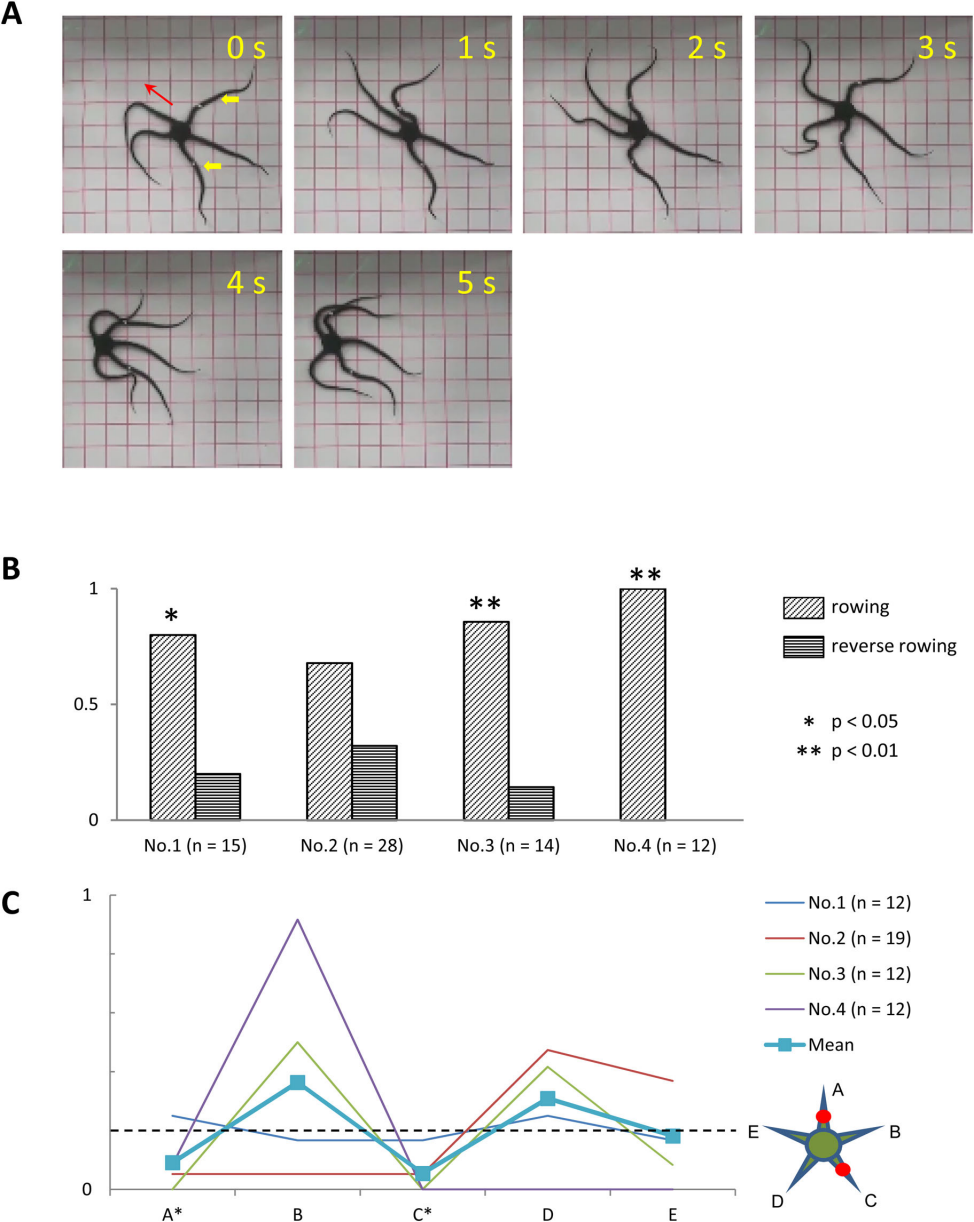
**Effects of anesthesia of two separate arms.** (A) Rowing movement of a subject (*O. incrassata*) in which the middle segments of two separate arms were anesthetized (indicated by yellow arrowheads). (B) Frequency of rowing and reverse rowing movements in subject Nos. 1–4. (C) Frequency at which each arm acted as the leading arm. Anesthetized arms are designated A and C and intact arms are designated B, D and E. Arm B is between A and C, whereas D and E are adjacent to C in a clockwise direction. Other conventions are the same as in Fig. 6.

We again found that the anesthetized subjects were able to coordinate arm movements for locomotion. Moreover, despite the fact that the anesthetized arms (which worked as forelimbs) separated the leading arm from the hindlimbs, coordination between these two entities was preserved (Fig. 9A). Although both types of movement were observed in anesthetized subjects, rowing movements were performed more frequently than reverse-rowing movements (Fig. 9B). While rowing, an intact arm functioned as the leading arm more frequently than an anesthetized arm. With the exception of just one subject, anesthetized arms acted as leading arms below chance level (Fig. 9C).

#### Anesthesia of four arms

**Fig. 10.**
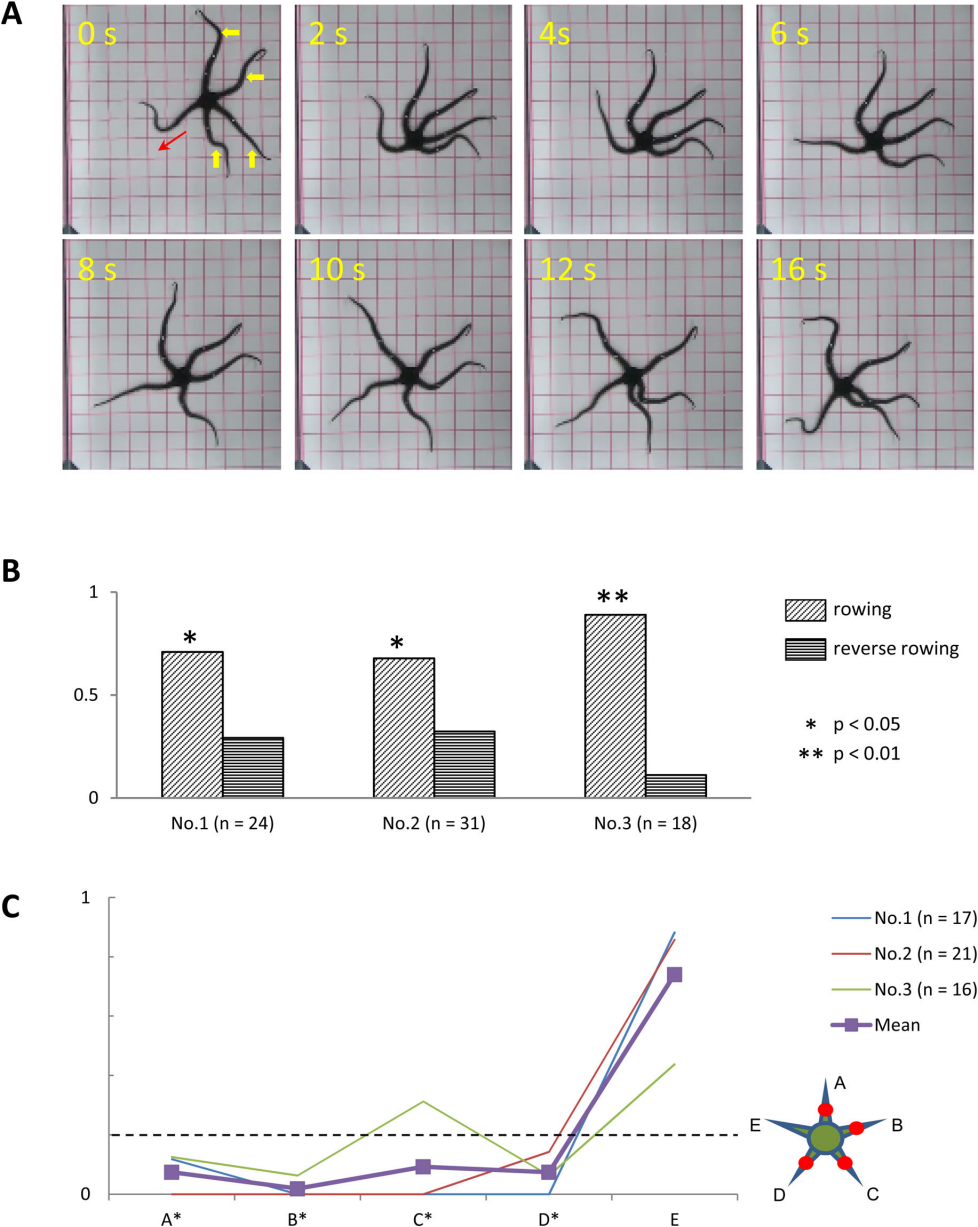
**Effects of anesthesia of four arms.** (A) Rowing motion of an subject (*O. incrassata*) in which the middle segments of four arms were anesthetized (indicated by yellow arrowheads). (B) Frequency of rowing and reverse rowing movements in subject Nos. 1–3. (C) Frequency at which each arm acted as the leading arm. Anesthetized arms are designated A–D, whereas the intact arm is designated E. Other conventions are the same as in Fig. 6.

We examined the effects of anesthetizing four arms in three subjects (two *O. incrassata* and one *O. superba*) to determine whether locomotion is still possible if the intact arm has no other intact arms with which to coordinate. All subjects were able to locomote following anesthesia (Fig. 10A) and preferentially performed rowing over reverse rowing movements (Fig. 10B). In addition, subjects used the intact arm as the leading arm for the majority of rowing movements (Fig. 10C). The intact arm functioned as the leading arm in 88% and 86% of all cases of rowing movements in both *O. incrassata*. In the *O. superba*, an anesthetized arm next to the intact arm functioned as the leading arm in 67% of all rowing movements and the intact arm functioned as the leading arm in 33% of all cases.

## DISCUSSION

### Functional localization in the ophiuroid nervous system

Previous studies have reported that echinoderms have non-centralized nervous systems. Morphologically, their nervous system lacks specialized nerve ganglia that are similar to the brain in other species such as vertebrates, cephalopods, and arthropods (Cobb and Stubbs, 1982). Physiological studies that have examined neuronal responses to photic stimulation reported that the circumoral nerve ring simply relays neuronal activity between radial nerve cords (Stubbs, 1982; Moore and Cobb, 1985). In addition, the nervous system of echinoderms reportedly has no specialized structures with which they can coordinate behavior; however, any part of the radial nerve cord can initiate locomotor behavior of the entire subject (Cobb, 1987, 1988, 1990, 1995).

As anticipated, ophiuroids in the present study demonstrated remarkable resilience even after the administration of nerve blocks. They were capable of performing stereotypical arm movements even after multiple parts of their body was anesthetized. This observation is consistent with the notion of a non-centralized nervous system, in that dysfunction of one part of the system is compensated for by the other intact parts.

Our experiments also demonstrated that the nervous system of ophiuroids features functionally localized organization. Anesthesia of the circumoral nerve ring completely disabled any initiation of locomotion. This effect was not due to dispersion of the anesthetic through the water vascular system to the radial nerves, because anesthetized subjects retained the ability to retrieve food with their arms (Fig. 3A; Movie 1). The varying effects of nerve blocks on various parts of the arm suggest that the radial nerves might also be functionally divided. Damage to the proximal radial nerves completely blocked swinging movements of the affected arm (Fig. 3B). Anesthesia of middle segments impaired inter-arm coordination, whereas spontaneous swinging movements of the affected arm were preserved (Fig. 4). Altogether, our findings suggest that although there is no evidence of a morphological counterpart to the central nervous system of other species, functional localization also exists in the nervous system of ophiuroids.

### Circumoral nerve ring

The present results suggest the importance of the circumoral nerve ring in locomotion. Anesthesia of the circumoral nerve ring completely disabled any initiation of locomotion. It may be argued that immobility was caused by diffusion of the anesthetic to the arms through the radial canals. This interpretation could be ruled out, however, because anesthetized subjects were able to grasp and relay food to the disc with their arms (Fig. 3A; Movie 1). This observation also suggests that food retrieval is performed by a series of local reflexes of the radial nerve that are independent of the circumoral nerve ring. An electron microscopic study indicated that the circumoral nerve ring (like the radial nerves), consists of ectoneural and hyponeural tissues (Cobb and Stubbs, 1982). Ectoneural tissue projects to the teeth, tube feet, gut, and peripheral regions of the arm, disc, and gonads. Hyponeural tissue projects mostly to the muscles (radial, external, and internal interradial muscles). The anesthetic agent used in the present study (L-menthol) likely anesthetized these diverse neuromuscular organs. In addition, a study on nerve degeneration revealed that the proximal radial nerve ganglion sends giant nerve fibers into the circumoral nerve ring (Cobb and Stubbs, 1981). It is also likely that anesthesia of the disc anesthetized the proximal ganglionic nerve fibers as well as their cell bodies. Given the fact that damage to the proximal radial nerve rendered the affected arm immobile, it is likely that either the circumoral nerve ring or the proximal radial nerve ganglia would have been responsible for the initiation of rhythmic movements of the arms during locomotion.

### Importance of contact stimuli for arm coordination

The rhythmic arm movements of ophiuroids during locomotion have been reported in previous studies (Arshavskii et al., 1976a; Astley, 2012). The arms and disc sequentially make contact with the floor during both rowing and reverse rowing movements. During the forward swing of the forelimbs, the arms are off the floor, while the disc remains on the floor. During the backward swing of the forelimbs, arms are in contact with the floor, while the disc is lifted and propelled forward (Fig. 2). The present study also suggests that the restricted area, rather than the entire arm, supports the disc during forward propulsion. Such movement would generate periodic on and off sensations as contact of the arms and disc is made with the floor.

Anesthesia applied to the midsegments of the arms blocked the sensations of periodic contact with the floor. This inference is supported by the unresponsiveness of the anesthetized portion to tactile stimulation. Total blockade of sensation in all arms completely disabled inter-arm coordination (Fig. 4A; Movie 2); however, motility of individual arms was preserved. These findings suggest that the sensation of contact (such as occurs against the floor) through the radial arms is critical for inter-arm coordination. It should be noted, however, that the arms that were cut proximal to the midsegment were still able to perform rowing movements (Fig. 5A,B). This suggests that ophiuroids can make use of the sensation of contact in various parts of their arms. It is also noteworthy that subjects with only one intact arm were still able to coordinate arm movements while rowing (Fig. 10). This finding suggests that any arm can compensate for the lack of sensation in other arms. Altogether, these findings allude to distributed and functionally resilient organization of the nervous system in ophiuroids.

### Functional localization within the radial nerve

The present study demonstrated that the effect of the radial nerve block depends on its anatomical location. Anesthesia of distal sections of the radial nerves induced no visible effects on locomotion. When the middle sections of radial nerves were anesthetized, the anesthetized arm rarely functioned as the leading limb while rowing. In addition, when all arms were anesthetized, the subjects were unable to coordinate arm movements. Nevertheless, when one or more arms remained intact, the anesthetized arms still performed swinging movements when functioning as forelimbs (Figs 8A, 9A and 10A), indicating that coordination among the intact segments was preserved.

Intra-arm coordination was completely abolished, however, by pinpoint damage to the proximal radial nerve (Fig. 3B). The arms with nerve damage exhibit no coordinated movements among the segments, but instead remained motionless during locomotion. This could not be attributed to rigidity of the arm induced by nerve damage because the lesioned arm still retrieved food that made contact with its distal areas.

These findings suggest that the radial nerve of ophiuroids is functionally diffuse yet nonhomogeneous: any part of the radial nerve can transmit the sensations necessary for locomotion (Fig. 5) whereas the proximal portions of the radial nerve are responsible for intra-arm coordination of the segments which generates rhythmic arm movements during locomotion. The latter interpretation is in agreement with the finding that a single amputated arm with an intact nerve ring can still produce autonomous rhythmic movements (Arshavskii et al., 1976b). Consistent with that report, when the arms were amputated leaving only the proximal portions, the operated subjects performed rowing movements with the arm stubs that remained (Fig. 5).

### Internal algorithm to determine the functions of the arms

In agreement with previous studies (Arshavskii et al., 1976a; Astley, 2012), the intact ophiuroids preferred rowing movements, otherwise referred to as the ‘breast stroke’ (Arshavskii et al., 1976a) over reverse rowing movements (Fig. 1). Nerve block experiments in the present study demonstrated that this preference for rowing was preserved even in subjects with multiple anesthetized arms (Figs 6, 7, 8, 9 and 10). In these experiments, the subjects generally preferred to use the non-anesthetized arm as the leading arm. Assuming that the nervous system of ophiuroids uses the functionally intact arm for the most critical role in locomotion, we propose that selection of the leading arm by the nervous system is a primary determinant in the assignment of various roles to the arms. Two previous studies are specifically relevant to our findings and interpretations. Arshavskii et al. (1976a) observed that the “end of the leading arm performed small searching movements”. Consistent with this observation, Astley (2012) suggested that the leading arm is used for sensory exploration. Our finding that anesthetized subjects primarily used the intact arm as the leading arm is consistent with these studies, because the anesthetized radial nerves would not have been able to convey sensory information received through its tip to other parts of the nervous system.

During locomotion, forelimbs either predominantly or exclusively contribute to propulsion (Astley, 2012). Nevertheless, the selection of forelimbs is unlikely a primary determinant in the control of locomotion. In several experiments, we left an even number of adjacent arms intact (Figs 6, 8 and 9). For example, anesthesia of three adjacent arms left two adjacent arms intact. If the nervous system selected the intact arms to propel the body most efficiently, the subjects should have performed reverse rowing movements using the two adjacent intact arms as a pair of forelimbs. In contrast, these subjects still preferentially performed rowing movements (Figs 6B, 8B and 9B). Therefore, the selection of forelimbs is unlikely to take priority over that of the leading arm.

### Methodological considerations

In the present study, we administered local nerve blocks to ophiuroids to determine the underlying neural mechanisms of locomotion. In various species, the control scheme of locomotion depends on control by both the nervous system and other mechanical properties of the body (Full and Koditschek, 1999). Amputation experiments of previous reports (Arshavskii et al., 1976b) would thus represent deviations from these qualitatively different locomotor mechanisms. The nerve block employed in the present study had an advantage over surgical measures in that it selectively affected the neuronal mechanism of locomotion, while preserving the body's mechanical properties (such as weight and center of mass). Also, anesthesia does not cause tissue damage that leads to production of inflammatory substances. This avoids the risk of inducing autotomy and reaction of mutative collagenous tissue that would again affect the dynamics of the body.

The effect of L-menthol in the present study should be interpreted with caution. Though the physiological effect of L-menthol on neurons has been studied in the vertebrate nervous system (Gaudioso et al., 2012; Eccles, 1994), its effect on various types of neurons in echinoderm remains to be studied. The nervous system of ophiuroids consists of ectoneural and hyponeural tissue. The ectoneural tissue innervates both sensory receptor cells and muscles, whereas the hyponeural cells exclusively innervate muscles and mutable collagenous tissue (Stubbs and Cobb, 1981; Heinzeller and Welsch, 2001). Furthermore, radial nerve ganglia consist of diverse types of neurons, which differ in terms of electrophysiological properties (Tuft and Gilly, 1984; Yee et al., 1987), mode of synaptic transmission (Yee et al., 1987), and neurotransmitters (De Bremaeker et al., 2000). The anesthetic (L-menthol) used in the present study is likely to indiscriminately inactivate these neurons. Further, because menthol passes through human skin and anesthetizes radialis nerve (Wasner et al., 2004), it is likely to have infiltrated to the circumoral and radial nerve in ophiuroids through the exoskeleton that is more permeable than human epidermis. In the process, the peripheral neurons in the superficial tissues may also have been anesthetized. Yet the present study indicated that the various parts of ophiuroids nervous system contribute differently to the initiation and coordination of arm movements. Further detailed functional neuroanatomy that underlies locomotion would be clarified by selective inactivation of specific neuronal populations in echinoderms.

## MATERIALS AND METHODS

### Subjects

We used the green brittle stars *Ophiarachna incrassata* (de Lamarck, 1816) whose arm length was between 10 to 15 cm, and the banded brittle stars *Ophiolepis superba* (Clark, 1915) whose arm length was between 8 to 15 cm in this study. All the experiments were conducted in accordance with the institutional guidelines of the Tohoku University. The ophiuroids were obtained from a commercial supplier (Nashville Co. Ltd., Miyagi, Japan) and kept in a laboratory tank filled with artificial seawater (density: 1.024–1.026 g ml^−1^, temperature: 24–25°C). The seawater was periodically replaced and constantly aerated by a protein skimmer, which also removed pollutants.

For each behavioral experiment, we randomly selected subjects with five intact arms of approximately equal length. Inactive or autotomized subjects were not used for the investigation. The subjects that autotomized their arms during behavioral observation were excluded from the subsequent statistical analysis. For behavioral observation of intact subjects, we used six subjects of each species. For nerve block experiments, two *O. incrassata* were used for disc anesthesia, three for anesthesia of all the arms, and three of each species were used for anesthesia of selected arms.

### Nerve block treatment

We used L-menthol (Serachem Co. Ltd, Hiroshima, Japan) for nerve block experiments in which we examined the roles of the circumoral nerve ring and radial nerves in the regulation of locomotion.

Prior to the behavioral experiments, we tested the anesthetic effect of L-menthol on ophiuroids to determine the time needed for anesthesia and recovery. We placed subjects in artificial seawater saturated with powdered L-menthol. Both species of subjects (*O. incrassata* and *O. superba*) were completely immobilized after 5 min of exposure to the anesthetic. When replaced in plain artificial seawater, recovery occurred within 30–60 min. The arms of anesthetized subjects were not autotomized during either the anesthetic or the recovery periods. Therefore, we considered that exposure to L-menthol for 5 min was sufficient to anesthetize ophiuroids, and proceeded with behavioral experiments. Surgical ablation of the nervous system was also tested. However, the subjects frequently autotomized the arms following surgery, thus we did not use this method as a standard measure of nerve block.

The subjects selected for nerve block experiments were taken out of water, then one arm of each subject was marked by white paint as a reference point. Then we applied powdered L-menthol to the selected part of the body for 5 min. The menthol powder was applied either to disc to anesthetize circumoral nerve ring, or to arms to anesthetize radial nerves. During anesthesia, we covered the menthol powder by elastic vet-wrap bandage (3M Japan, Tokyo, Japan) to prevent its dispersion to other parts of the body. To prevent dehydration, artificial seawater was applied using a medicine dropper to the areas in which the anesthetic was not applied.

Following the required duration of anesthesia, the subjects were washed with artificial seawater to remove all traces of the menthol, after which the subjects were returned into sea water and the behavioral experiments commenced. At least one week of interval was given to the anesthetized subjects before they were re-used for other experiments.

In cases that required finely circumscribed blockade of the radial nerves, we anesthetized the selected portion of the arms with L-menthol for 5 min, and using a fine drill bit (diameter: 0.5 mm), made a small opening in the oral arm shield of the anesthetized portion that was extended to the epineural sinus. Then, lesions were inflicted on the radial nerve running along the aboral wall of the sinus (Cobb and Stubbs, 1981) by the same drill bit. The subjects that underwent surgical nerve ablation were not re-used in any subsequent experiments.

### Monitoring of locomotion

After the nerve block, the subjects were placed in a tank that measured 30×30 cm. The tank was filled with artificial seawater and its conditions (density and temperature) were maintained at levels comparable to those of the holding tank. Aeration was conducted for 10 min before any subject was placed into the tank. The subjects were then placed in the center of the tank and allowed to move freely. Intact ophiuroids naturally avoid open spaces, therefore when placed in the center of the tank, they immediately moved to a corner. Each time a subject was placed in the center of the tank, its body was rotated to randomize the spatial relationship between the arms and any potential environmental cues (e.g. shadows or tilt). While moving towards the corners, the movements of the subjects' arms were recorded by a digital video recorder (HDR-XR150, Sony, Tokyo, Japan) at 30 frames per second with a resolution of 1280×720 pixels, and saved for subsequent analysis. Each behavioral experiment lasted for 30 min because, based on the above described preliminary test of anesthesia, the effect of L-menthol was considered to last for at least 30 min. During this period, we monitored their locomotion and classified its type. For locomotion that was identified either as rowing or reverse rowing, we recorded the arm which acted as the center limb.

### Data analysis

In the present study, we focused our analysis on rowing and reverse rowing. We counted the occurrences of these two types of movements, and examined the roles played by intact and anesthetized arms during the locomotion. The behavioral data from the selected subjects were used for statistical analysis only when both of the two following criteria were met. First, we chose the subjects for which the combined sum of occurrences of rowing and reverse rowing exceeded 10 during the observation period (30 min). Second, we used the data from subjects that did not autotomize the arms during the observation. If the number of the locomotion did not exceed 10 or if the subjects autotomized any of the arms during the experiment, such subjects were excluded from the analysis. A two-tailed chi square test was performed to examine whether there was a bias in the occurrences of rowing and reverse rowing. We also examined whether the roles played by intact and affected arms in locomotion were also biased.
